# Within- and between-person changes in work practice and experiences due to COVID-19: Lessons learned from employees working from home, hybrid working, and working at the office

**DOI:** 10.3389/fpsyg.2022.948516

**Published:** 2022-12-21

**Authors:** Siw Tone Innstrand, Marit Christensen, Karoline Grødal, Cristina Banks

**Affiliations:** ^1^Department of Psychology, Faculty of Social and Educational Sciences, Norwegian University of Science and Technology, Trondheim, Norway; ^2^School of Public Health, University of California, Berkeley, Berkeley, CA, United States

**Keywords:** COVID-19, home office, MANOVA, gender difference and similarity, occupational health, generalized linear models (GLMv)

## Abstract

**Introduction:**

In response to the requirement of keeping social distance during the COVID-19 outbreak a lot of employees needed to change from a regular office to a home-office at short notice. The aim of the present study is to explore these employees' experiences and evaluate changes in their work situation during the pandemic.

**Method:**

A mixed-method design was used with panel data collected twice in an insurance company in Norway. The first dataset was collected in December 2020 (Time 1; *N* = 558), with a follow up in March 2021 (Time 2; *N* = 601).

**Results:**

Our study indicated that employees' main reasons for working from home were to keep social distance, avoid contagion and protect their loved ones. Flexibility, timesaving and more time with family and friends were also motivators. Most employees reported that they had the necessary technical equipment to work from home and wanted more opportunity to use their home office in the future. General Linear Models (GLM) indicated that work-family balance and workload were the same across age, gender, and worksites. Women and employees working from home reported more fear of being infected by COVID-19 at work. Younger employees reported experiencing less social contact with colleagues than normal during the pandemic, compared to the older employees. Overall, employees working at home were more positive toward digital solutions and digital meetings than those at the office. Repeated measures MANOVA showed that the work motivation and digital competence decreased over time for all worksites. Productivity increased for home-office employees but decreased for the hybrid and work-office employees.

**Discussion:**

This paper contributes to knowledge of employees' experiences with different worksite solutions, which will be useful for anticipating employees experience in the future with more hybrid work.

## Introduction

Until recently, most work arrangements have been ingrained by the Industrial Revolution with employees mainly transacting their time rather than their output with employing firms. For many employees, physical attendance at the workplace, employing a “nine-to-five” working style, has been the expectation (Gajendran and Harrison, 2007). Although the information revolution with digital technologies has compelled firms *to* provide an increased allowance for remote working, this trend was accelerated by the outbreak of the COVID-19 pandemic. As a measure of keeping social distancing, occupational groups with non-essential functions were asked to work from home with an increased risk of isolation, loneliness, technostress, and work–family conflict (Tuzovic and Kabadayi, 2021). As a result, almost 40% of paid work among EU member states was carried out at home during the pandemic (Eurofound, 2020).

The pandemic is predicted to have both a short-term and long-term impact on society, health care, jobs, and individuals (Burdorf et al., 2020). Although the acute need for teleworking (performed work at an approved alternative worksite) may diminish as the pandemic comes under control, the major impact on how we think and act about work and working life will remain (Kniffin et al., 2021). Previous pandemics such as the Black Death in 1347–1353, the Spanish flu in 1918, and more recently SARS in 2003 also affected how working life can be understood, carried out, and organized (Rudolph et al., 2021). According to event systems theory, all changes should be considered by how prominent an event is (Morgeson et al., 2015). The more unusual and novel (unknown), disruptive, and critical an event is, the more likely it is to change or create new behaviors, functions, and events. Since the outbreak of COVID-19 has proven to be a completely new, global catastrophe that affected entire societies in many ways, there is reason to believe that some changes will endure. Also, it has been suggested that “extreme events” such as the onset of COVID-19 often provide a window to identifying and understanding important dynamics that are not necessarily visible under normal conditions (Kniffin et al., 2021).

This study aims to improve our understanding of the impact of COVID-19 on work life by simultaneously examining between-person and within-person effects of COVID-19 experiences and job changes using panel data from an insurance company in Norway. Specifically, the aim of this study was 3-fold: (I) to explore conditions for working from home (reasons for working home, home-office facilities, and future perspectives for working from home by using both quantitative and qualitative data); (II) to identify group differences (gender, age, and worksite) in participant experience regarding how COVID-19 has affected their work lives; and (III) to investigate within-person and between-person changes in the evaluation of how COVID-19 has altered different aspects of work across different work sites. Based on the objectives of the study and previous findings and theories, the following assumptions are stated: (1) reasons for working from home, home-office facilities, and future perspectives for working from home will remain stable between Time 1 and Time 2; (2) participants will differ in their COVID-19 experiences with regard to demographic characteristics; and (3) participants will experience some between-person (home office, hybrid, and workplace) and within-person (time) job changes during the COVID-19 pandemic.

### Working from home

While home office refers to work tasks performed at home, telework is a broader term that indicates work that can be done anywhere outside the physical workplace (Kniffin et al., 2021). There is a considerable amount of research exploring home offices before the pandemic, suggesting less work-to-home conflict but more home-to-work conflict on teleworking days compared to non-teleworking days (Delanoeije et al., 2019). However, the reasons for working from home during the pandemic, and hence the consequences, might differ from previous studies. Working from home was, for many employees, required with short notice by the employer or the government in response to the need to keep social distance and not a voluntary action to balance work and family life (Tuzovic and Kabadayi, 2021). Thus, many did not have time to prepare for or facilitate these changes. As suggested by Delanoeije et al. (2019), this lack of control, together with a lack of time to adapt to this new situation (i.e., time to arrange for decent home-office facilities, new routines with balancing home and family life, and technological skills), might have influenced how telework was experienced by employees.

A review by Como et al. (2020) shows that although many report increased workload and overtime working from home, others enjoy the flexibility of being able to plan their own working day, fewer interruptions from colleagues, and reduced commuting time. Based on a review of the literature and focus group interviews, Ingusci et al. (2022) identified seven benefits (i.e., Economical and/or time-saving in traveling) and seven disadvantages (i.e., Reduced visibility toward superiors and/or recognition of own work) related to remote working. This aligns with a longitudinal study by Pirzadeh and Lingard (2021) who found both positive and negative experiences of home-based teleworking during the COVID-19 pandemic, but the negative outweighed the positive over their seven waves of the study. Different experiences with home offices are explained by differences in office facilities, resources at home, and family situations (Como et al., 2020). For example, the findings of Vaziri et al. (2020) suggest that changes in work and family balance during the COVID-19 pandemic can be explained by both personal and work factors such as coping style, technostress, and leadership style. Technostress might be especially challenging for older workers (Nimrod, 2022). This indicates that there might be different determinants of successful work from home and a possible differential impact on employee health and well-being.

It has been suggested that the future of work is here, and it is hybrid, meaning that flexible work is here to stay (Microsoft, 2021). Eurofound's study (Eurofound, 2020) indicates that three out of four workers in the EU Member States reported that they would prefer to work from home, at least occasionally, even if there were no COVID-19 restrictions. Corresponding figures from the US show that 63% of employees want to work fully or partly at home after the pandemic (Alexander et al., 2021b) and that nine out of ten organizations want to combine teleworking with a physical presence at work (Alexander et al., 2021a).

The present study aims to explore insurance workers' experience of working from home during the pandemic in Norway. Specifically, the study aims to explore the following:

*Research question 1:* What do the respondents report as reasons for working from home, to what extent do they have access to home-office facilities, and what is their desire for working from home in the future?

### Group differences in the experience of COVID-19

It has been argued that the impacts of the pandemic will affect some groups of workers more strongly than others based on their age, race and ethnicity, gender, or personality (Kniffin et al., 2021). For example, differences in socioeconomic status could be a source of considerable disparity for population segments that had limited (or null) access to devices that ensured constant connection with their work and social environment. According to the Vitamin model (Warr, 2017), any environmental characteristic (i.e., work–home conflict, workload, social contact, and safety) must be considered in relation to within-person differences. These within-person sources might be longer-term characteristics such as dispositional or demographic features, or short-term, situation-based mental processes. The latter relates to different assessments of novelty or familiarity (i.e., to what extent is the situation unusual or previously experienced), comparison with our situations, future trends anticipated, or personal salience/value of the situation (i.e., “how much do I want to be in this role”). The requirement to socially distance to avoid the contagion of COVID-19 has provided a new, and for some an inadvertent, working situation for a lot of employees. The present study explores how environmental characteristics related to COVID-19 experiences at work differ both according to fixed characteristics (gender and age) and situation-based processes (different worksites).

Morales-Vives et al. (2020) explored sociodemographic variables related to adapting to lockdown in a large Spanish population. They found that sex and age especially should be considered in understanding COVID-19 impact. Their study indicated that women tended to show greater stress, lower self-esteem, and a more pessimistic attitude about the lockdown due to worrying more about the health crisis, being more afraid of themselves or relatives getting infected, and talking more about the disease. Older people were also more worried but adapted better to the lockdown. This relates to the findings from Al Miskry et al. (2021) that showed more distress, worry, avoidance, and emotion-focused coping during the pandemic experienced by women. With respect to age groups, their findings suggest more distress and worry among the young (22–29 years). In general, research indicates that the pandemic had a more severe effect on the mental health of women and young people (Fenollar-Cortés et al., 2021; Salazar et al., 2021). According to Salazar et al. (2021), these findings might be explained by a pre-COVID-19 vulnerability in these groups with women being more sensitive in general and higher rates of mental problems among the young due to job insecurity and worries about the future, which has only been accelerated by the pandemic.

A recent report by Microsoft (2021) suggests that Gen Z (those between the age of 18 and 25) is a highly overlooked demographic group that appears to be suffering a lot because of the pandemic. Their annual Work Trend Index from 2021 based on responses from more than 30,000 people from 31 countries indicates that Gen Zs are more likely to struggle to balance work with life and to feel exhausted after a typical day of work compared to older generations. As suggested by their findings, Gen Zs are more likely to be single, making them more vulnerable to the impact of isolation. Moreover, being early in their career they are more likely to lack the financial means to create proper workplaces at home.

Eurofond's study (2020) showed that women especially found it difficult to concentrate on work and balance work and family life while working from home. This is consistent with recent findings reported by Hayes et al. (2021), which suggest an increase in perceived stress under COVID-19 restrictions, especially for women and for people with limited experience working from home. One study claims that the pandemic has led to a step back to traditional gender roles among men and women, where women have taken on most of the responsibility of the family during the lockdown (Lewis, 2020).

In general, there is a lack of research comparing employee health and well-being working from home vs. the workplace, including the need for organizational support and the impact of the collective trauma COVID-19 brought (Como et al., 2020). Preliminary research indicates that people who worked from home during the pandemic have higher levels of work-related burnout (Hayes et al., 2021), report a lower probability of feeling that they are doing a useful job, have more quantitative requirements, and feel more isolated compared to those who worked from the workplace (Eurofound, 2020). Yet, we do not know how different work arrangements might have affected job-related experiences due to COVID-19 during the pandemic.

In sum, to shed light on demographical differences in mental health reported during the pandemic, the present study explores how job-related experience due to COVID-19 (i.e., work–home balance, attitudes toward digital solutions, and workload) might differ across gender, age groups, and different worksites (home office, hybrid, and workplace).

*Research question 2:* How will employees' job-related experience due to COVID-19 differ across gender, age, and worksites?

### Within-person change

Previous studies on telework have been largely inconclusive (Delanoeije and Verbruggen, 2020); some suggest favorable outcomes (i.e., enhanced work engagement, lower stress, and increased job performance), others suggest no effects, and others suggest unfavorable outcomes such as more stress. Delanoeije and Verbruggen (2020) suggest that these inconclusive findings might be explained by differences in content, context, and methodological-related explanations like how and why telework was implemented (i.e., trust from leader), family situation (i.e., risk of boundary blurring), and selection effects (i.e., higher ex-ante levels of stress or work–home conflict among tele-workers). Another explanation might be due to the dominant approach of studying only between-person effects between users-and non-users of telework, overlooking the potential within-person effect over time. Thus, the present study aims to explore both within-person (time) and between-person differences (worksites) in respondents' evaluation of change due to COVID-19.

In line with the Vitamin model (Warr, 2017), research has suggested that positive feelings in response to an environmental stimulus can be reduced or even give way to indifference over time due to adjusting to the situation. For example, adjustment to the situation may indicate that the happiness or unhappiness of people whose situation is improved or deteriorated is likely to return to the equilibrium level. This kind of “honeymoon effect” has also been reported during the pandemic (Pretus and Vilarroya, 2022). Thus, any increase in motivation, workload, or similar reported at the beginning of the pandemic can be constant or even reduced as the novelty or excitement of the situation wears off or a person adjusts to the situation. Indeed, in a review, Domenicano (2020) found that, despite severe negative psychological reactions reported during the pandemic, some studies observed a decrease in negative emotions such as fear and anxiety in caregivers after the peak of the outbreak and after work adjustments took place. Comparing data before and after telework was introduced, Delanoeije and Verbruggen (2020) found a decrease in stress at Time 2, but no change in work engagement, work–home conflict, or job performance. Similarly, Oksa et al. (2021) found that work engagement remained relatively stable during their four-wave study during the COVID-19 crisis, and only decreased slightly at Time 4 (in autumn 2020). A multiwave survey among construction workers working from home in Australia (Pirzadeh and Lingard, 2021) found a consistent gradual decline in the mental well-being among the participants; however, they did not find any significant differences in well-being across worksites (working from home or the office).

The present study aims to explore both within-person (time) and between-person effects (worksites) of respondents' evaluation of change in their working situation due to COVID-19. Specifically, we hypothesize the following:

*Research question 3:* Will job-related change variables (work motivation, work quality, work amount, concentration, contact with colleagues, digital competence, and leader support) remain stable, or slightly return to equilibrium between the two time points as the employees adapt to the situation?

*Research question 4:* Will the respondents' perceptions of how their work situation has been impacted by the pandemic differ across different worksites (home office, hybrid, and at the workplace)?

## Methods

### Norway as a context

On 12 March 2020, the most intrusive measures Norway has seen since World War II were implemented. Among other things, the country's schools and kindergartens closed down, as did a number of companies and service industries. The shutdown led to an immediate massive increase in unemployment and layoffs, and hundreds of thousands worked from home, with either a home office or homeschooling. The use of public transport and driving decreased sharply. After several waves of the peak in the contagion, with varying infection control measures, the national measures and most local measures were repealed on 24 September 2021 with effect from the next day. A new wave led to new measures, which were then repealed on 12 February 2022.

### Participants

The present study uses longitudinal panel data collected twice during the COVID-19 pandemic from an insurance company in Norway. The insurance company was part of a larger cross-Atlantic study (Norway and USA) on healthy workplaces in 2019, and when the pandemic hit a few months later it was decided to make a follow-up for this company. The present study is based on this follow-up. The first dataset was collected in December 2020, with a follow-up in March 2021, at a time when parts of Norway entered a major social lockdown as the number of COVID-19 infections started to rise again. All employees in an insurance company (*N* = 1,400) were sent an email including a link to a survey and an information letter about the purpose of the surveys with study details including contact information and assurance of confidentiality, anonymity, and voluntary participation. A reminder was sent out a week later and the link was open for 3 weeks. A personal identification code made by the respondents allowed us to track the same individuals through the subsequent surveys over time.

Time 1 dataset (*N* = 558) consisted of 287 (51.4%) women and 268 (48%) men, providing a 40% response rate. One respondent identified himself as non-binary and two respondents did not want to state their gender (0.6%). Age was distributed with least employees (9.7%) in the youngest category (< 30 years), most employees aged 31–50 years (45.5%), closely followed by those 51 years or older (44.8%). A total of 40.5% reported 3 years of higher education, and 24.2% had 5 or more years of higher education. The rest had high school or less. Most employees had a permanent position (99.5%) and worked full-time (92.3%).

Time 2 data (*N* = 601) had a response rate of 43% and aligns with Time 1 data regarding the distribution of gender [women (50.2%), men (49.8%)] and age [<30 (10.1%), 31–50 (44.8%), 51 years or older (45.1%)]. Forty-one percent reported 3 years of higher education, and 22.1% had 5 or more years of higher education. Most employees had a permanent position (99.3%) and worked full-time (92%).

Table 1 provides the frequency of employees working from home, with a hybrid solution, or who were physically at the workplace before COVID-19 and at Time 1 and Time 2. The before-COVID-19 figures are based on retrospective questions measured at Time 1. The majority reported that they were physically present at the workplace before the pandemic (75.8%), whereas most had a home office at Time 1 (62%) and Time 2 (76.4%).

**Table 1:** Distribution of employees responding on which alternative best describes their working situation before and during (Times 1 and 2) COVID-19.

	**Pre-Covid-19** ^*^		**Time 1**		**Time 2**	
	**Frequency**	**Valid percent**	**Frequency**	**Valid percent**	**Frequency**	**Valid percent**
Home office	17	3.0	346	62.0	459	76.4
Hybrid	118	21.1	136	24.4	90	15.0
workplace	423	75.8	76	13.6	52	8.7
Total	558	100.0	558	100.0	601	100.0

* Retrospective question measured at Time 1.

### Variables

*Gender* was measured using five different response options: (1) ≪*Woman*≫ (2) ≪*Man*≫ (3) ≪*Non-binary*≫ (4) ≪*Other*≫ and (5) ≪*Do not want to enter*≫ at T1 and T2. Values three to five were omitted from the analyses, whereas the rest was dichotomized into (1) women and (2) men.

*Age* consisted of 105-year intervals from 16–20 to 61–65, in addition to a final answer option that included 66+. These questions were the same for T1 and T2. These were split into three categories for the analyses: young (31 years or younger), middle-aged (31–50 years), and older (51 years or older).

*Work situation* was measured by asking about the current work situation and retrospectively before COVID-19: “Which alternative gives the best description of your work situation the last month (current)/before COVID-19 (pre-COVID-19)?.” Both scored on a 3-point scale with the alternatives: (1) I am (was) working from home, (2) I (used to) work both from home and at the workplace, and (3) I go (went) to work every day. Both the retrospective and current work situations were asked at T1, followed up by only the current work situation at T2.

*Conditions for working from home* were measured at T1 and T2 by thirteen single items developed by Langvik et al. (2021) for their study on working conditions for policemen during the COVID-19 pandemic. The statements were themed together with six items related to reasons for working from home like “I work better from home,” five items related to home-office facilities like “My living situation is suitable for home office work,” and finally, two items asking about the desire to work from home like “Home office should be able to be used more.” The latter category was supplemented with two autonomy-related items at T2 (i.e., “I want to be able to choose to work from home in the future”). All items are listed in Table 2. All statements were responded to on a 5-point Likert scale ranging from “*totally disagree”* to “*totally agree.”*

**Table 2:** Frequency and valid percent for reasons for working from home, home office facilities and the desire for home office now and for the future at Times 1 and 2.

	**Time 1**		**Time 2**	
	**Frequency**	**Valid percent**	**Frequency**	**Valid percent**
**Reasons for working from home:** ***(disagree/neither/agree)***				
I work better from home	126/200/232	22.6/35.8/41.6	117/183/249	21.3/33.3/45.4
Infection control—I want to protect my loved ones	53/112/393	9.5/20.1/70.4	53/83/413	9.7/15.1/75.2
Infection control—I want to shield the workplace	54/132/372	9.7/23.7/66.7	48/87/414	8.7/15.8/75.4
It is a requirement from the employer/government^*^	69/114/375	12.4/20.4/67.2	13/47/489	2.4/8.6/89.1
It makes everyday life easier	89/116/353	15.9/20.8/63.3	88/111/350	16.0/20.2/63.8
To get away from environments in the workplace	430/90/38	77.1/16.1/6.8	447/68/34	81.4/12.4/6.2
**Home office facilities:** ***(disagree/neither/agree)***				
My living situation is suitable for home office work	75/63/420	13.4/11.3/75.3	79/77/445	13.1/12.8/74.0
I have access to necessary technical equipment	43/42/473	7.7/7.5/84.8	57/48/496	9.5/8.0/82.5
I can work undisturbed at home	56/47/455	10.0/8.4/81.5	53/44/504	8.8/7.3/83.9
I have access to the necessary facilitation at home	67/84/407	12.0/15.1/72.9	72/92/437	12.0/15.3/72.7
Home office works mainly well for me	61/73/424	10.9/13.1/76.0	74/73/454	12.3/12.1/75.5
**Desire for home office** ***(disagree/neither/agree)***				
Home office should be able to be used more in the future	47/79/432	8.4/14.2/77.4	42/72/487	7.0/12.0/81.0
I wanted to use a home office before	124/119/315	22.2/21.3/56.5	163/139/299	27.1/23.1/49.8
I want to be able to choose to work from home now	–	–	75/92/434	12,5/15,3/72,2
I want to be able to choose to work from home in the future	–	–	55/70/476	9.2/11.6/79.2

* “Required by the employer” changed into “required by the government” in T2.

In addition to these quantitative questions one open-ended, qualitative question was used in this study: “*Are there other reasons why you work from home*.” This qualitative question complemented the quantitative statements by providing more in-depth knowledge on the reasons why they were working from home, i.e., why it makes their life easier or how they work better from home. It also allowed for other reasons not covered by the quantitative questions.

*COVID-19 job experiences* were measured at T1 by seven single statements developed by Langvik et al. (2021) for their police study. All statements were responded to on a 5-point Likert scale ranging from “*totally disagree”* to “*totally agree.”* Sample item: “It is harder to balance work and family life during COVID-19.”

*Job changes due to COVID-19* were measured at T1 and T2 by seven single statements developed by Langvik et al. (2021) for their police study. The respondents evaluated to what extent different work characteristics such as their work motivation, quality of work, and amount of work getting done (productivity) had changed due to COVID-19 by using a five-point Likert scale: (1) *Very reduced*, (2) *Reduced*, (3) *Just like before COVID-19*, (4) *Increased*, and (5) *Increased significantly*.

### Statistical analysis

All statistical analyses were performed by using SPSS version 27. Conditions for working from home were assessed with both quantitative and qualitative data. First, “reasons for working home,” “home office facilities,” and “future perspectives for working from home” were measured by the quantitative conditions for working from home variables and categorized into three categories: disagree (“strongly disagree” and “disagree”), neither disagree or agree, and agree (“agree” and “strongly agree”). Frequency analyses were performed on all Time 1 and Time 2 variables.

These data were supplemented with additional information from the open-ended, qualitative question; “*Are there other reasons why you work from home*.” Following the steps by Nowell et al. (2017), nine themes were identified after reading and getting familiarized with the data and selecting comments relevant to the study. Those who only reported “I do not work from home” were removed. In total, 88 comments at T1 and 92 valid comments at T2 were received from the participants. The remaining comments aligned with the six themes used in the qualitative survey. In addition, three more themes were identified in the data: “Infection Control—I want to protect myself,” “Cultural pressure,” and “Worked from home pre COVID-19.” Some comments reflected more than one theme. The themes and the associated frequencies are presented in Table 3.

**Table 3:** Reasons for working home identified in participant's comments (qualitative, open-ended question).

	**Number of comments expressing the theme**	
**Reasons for working from home**	**Time 1**	**Time 2**
I work better from home	*N* = 16	*N* = 17
Infection control—I want to protect my loved ones	*N* = 4	*N* = 4
Infection control—I want to shield the workplace	*N* = 0	*N* = 0
It is a requirement from the employer/government	*N* = 19	*N* = 30
It makes everyday life easier	*N* = 30	*N* = 31
To get away from environments in the workplace	*N* = 1	*N* = 2
**In addition to the reasons measured quantitative**		
Infection control—I want to protect myself	*N* = 16	*N* = 4
Cultural pressure	*N* = 1	*N* = 1
Worked from home pre COVID-19	*N* = 1	*N* =3

Some comments reflected more than one theme.

Group differences in job experiences due to COVID-19 were analyzed by univariate General Linear Models (GLM) with the COVID-19 job experience variables as the dependent variable and gender, age, and worksite (home office, hybrid, and workplace) as fixed factors separately. Skewness and kurtosis values were computed to test the normality of the univariate distribution of the data. Skewness and kurtosis values were within the range of normality (±1.96) (Gravetter and Wallnau, 2014). Moreover, frequency analyses were performed by dichotomizing the job experience variables into high (“agree” and “totally agree”) and low (“totally disagree,” “disagree,” and “neither”). Only Time 1 data were used for these analyses.

To explore within-person and between-person change in the evaluation of work arrangement due to COVID-19 at Time 1 and Time 2, we performed repeated measures MANOVA in SPSS with time (i.e., dependent variables measured at Time 1 and Time 2) as the within-subject variable and worksite (home office, hybrid, and workplace) as the between-subject variable. In addition, we explored the interaction effects between time and worksite on the dependent variable. Skewness and kurtosis values were computed to test the normality of the univariate distribution of the data. Skewness and kurtosis values were within the range of normality (±1.96) (Gravetter and Wallnau, 2014) at Time 1 and Time 2. Two exceptions were found for “*The quality of work I do is”* (Kurtosis: 2.28) and “*Support from immediate manager is”* (Kurtosis: 2.32) which had a slightly too high Kurtosis at Time 1. However, these were within the range of normality at Time 2. The main hypothesis was that the COVID-19 impact variables would change between the two time points as the employees adapt to the situation. Moreover, we expected differences between the different worksites in how their work situation had been impacted by the pandemic. If significant, Bonferroni-adjusted pairwise comparisons will be used to detect where the differences are. Finally, we calculated partial eta-squared (η^2^) following the general rule of thumb by Miles and Shevlin (2001) that η^2^ = 0.01 indicates a small effect; η^2^ = 0.06 indicates a medium effect; and η^2^ = 0.14 indicates a large effect.

## Results

### Reasons for working from home

Table 2 provides the frequency and valid percent for reasons for working from home, home-office facilities, and the desire for home working now and for the future at Time 1 and Time 2. Regarding reasons for working from home, most agreed on “Infection Control—I want to protect my loved ones” (70.4%) and “It is a requirement from the employer” (67.2%) at Time 1. The lowest agreement was on “To get away from environments in the workplace” (6.8%). Similarly, at Time 2 most agreed on “Infection Control—I want to shield the workplace” (75.4%) and “It is a requirement from the government” (89.1%), whereas the least agreed point was “To get away from environments in the workplace” (81.4%).

Exploring home-office facilities, most respondents agreed that they “have access to necessary technical equipment” (84.8/82.5%) and “can work undisturbed at home” (81.5/83.9%) both at Time 1 and Time 2 (T1/T2). There seems to be a common agreement among the respondents at both T1 and T2 that they want to be able to work from home more in the future (77.4/81%).

The quantitative data on reasons for working from home were supplemented by findings from one open-ended question: “Are there other reasons why you work from home?.” In addition to the six categories given in the quantitative survey asking for reasons for working from home, three additional categories were identified in the open-ended question: “Infection Control—I want to protect myself,” “Cultural pressure,” and “Worked from home pre COVID-19” (refer to Table 3). In the qualitative analysis, no respondents indicated that the main reason for working from home was to avoid the workplace. Instead, most indicated that they work better from home (*N* = 16/17) and are following the requirement from the employer/government (*N* = 19/30), or because it makes their life easier (*N* = 30/31). Many reported that they worked better from home because they were more efficient, they get more peace to work, were less disturbed, more concentrated, had better and easier meeting facilities through Teams (=digital communication tool), and because it was better to have sensitive conversations with customers at home as compared to open offices. Other reasons such as better indoor climate and less noise were also reported. A better indoor climate was associated with fewer headaches and fatigue.

In addition, many felt that home working made their life easier because they did not have to commute and thereby saved time, the environment, and money for gas and wear of the car. It was also more practical with less stress associated with bringing and picking up children from school and taking care of children who were sick or needed to be at home. Some recently acquired a pet and said it was convenient when they were at home and could take care of it. In general, they felt they saved time and had more time with family and friends, more spare time, more time for exercise, and more time for restitution and sleep. Overall, they reported better quality of life, less stress, and a better balance between work and family life. The qualitative results complemented the quantitative analysis by providing more in-depth knowledge on the reasons why they were working from home, i.e., why it makes their life easier or how they work better from home. It also allowed for other reasons not covered by the quantitative questions. In particular, the desire to protect one-self and not to avoid the workplace was evident at Time 1.

### Gender, age, and worksite differences

Table 4 provides the frequencies, means, standard deviations (in italics), and results of the univariate GLM of COVID-19 experiences comparing gender, age, and worksite (home office, hybrid, and/or workplace). Altogether, 40.1% reported “totally agree” or “agree” that it was hard to cleanly separate between home life and work during COVID-19. The GLM analysis indicated that there were no age, gender, or worksite differences in this statement. Seventy-nine percent have become more positive about digital solutions. There was no significant difference in this attitude between gender and age groups, but there were significant differences across the work sites [*F*_(2, 558)_ = 7.69, *p* < 0.001, *η*_*p*_^2^ = 0.027). Multiple comparisons with Bonferroni indicated that those with a home office (*M* = 4.18) and those with a hybrid solution (*M* = 4.09) were significantly more positive to digital solutions than those who were at the office (*M* = 2.70). There were no significant differences between those working mainly from a home office and those with a hybrid solution.

**Table 4:** Frequencies, means, standard deviations (in italics), and results of the univariate GLM of COVID-19 experiences comparing gender, age, and worksite (home office, hybrid, and workplace).

**Covid-19 experience^a^**	**Frequencies^b^**	**Gender**		**Age**			**Worksite**		
	**Cumulative %**	**Women**	**Men**	**< -30**	**31–50**	**51 → **	**Home**	**Hybrid**	**Office**
Work-home balance during COVID-19	40.1%	2.86 *(1.38)*	2.88 *(1.30)*	2.87 *(1.44)*	2.96 *(1.34)*	2.78 *(1.34)*	2.95 *(1.36)*	2.84 *(1.32)*	2.59 *(2.21)*
Positive attitudes toward digital solutions	79%	4.09 *(0.88)*	4.11 *(0.77)*	4.15 *(0.10)*	4.16 *(0.78)*	4.04 *(0.83)*	4.18^*^(0.80)	4.09 *(0.81)*	3.78 *(0.90)*
Digital meetings have function well	86.6%	4.37 *(0.73)*	4.21 *(0.76)*	4.31 *(0.84)*	4.31 *(74)*	4.26 *(74)*	4.41^*^(0.73)	4.23^*^(0.72)	3.88 *(0.77)*
More workload during COVID-19	32.8%	3.18 *(1.10)*	3.08 *(0.98)*	3.37 *(1.00)*	3.09 *(1.10)*	3.13 *(1.01)*	3.17 *(1.06)*	3.05 *(1.01)*	3.14 *(1.01)*
Desire more for frequent use of home-office	74%	4.03 (1.21)	3.90 *(1.17)*	4.11 *(1.03)*	4.05 *(1.16)*	3.86 *(1.25)*	4.24^*^(0.98)	3.98^*^(1.05)	2.70 *(1.46)*
Fear of being infected by COVID-19 at work	36.9%	3.06^*^(1.26)	2.78 *(1.24)*	3.13 *(1.33)*	2.80 *(1.30)*	3.01 *(1.22)*	3.11^*^(1.26)	2.64 *(1.26)*	2.59 *(1.20)*
Less social contact than normal with colleagues outside work during COVID-19	64.3%	3.85 *(1.18)*	3.79 *(1.11)*	4.04^*^(1.18)	3.91 *(1.11)*	3.67 *(1.17)*	3.86 *(1.16)*	3.79 *(1.11)*	3.66 *(1.12)*

a See method section for a full description of the statements,

b Based on those responding agree or totally agree,

* Significant higher levels as indicated by the GLM analysis.

Digital meetings seem to have functioned well, as 86.6% agree or totally agree with this statement. However, the GLM analysis indicated that there were significant differences in the satisfaction of the digital meetings statement across the work sites [*F*_(2, 558)_ = 16.89, *p* < 0.001, *η*_*p*_^2^ = 0.057]. Multiple comparisons with Bonferroni indicated that those with a home office (*M* = 4.41) were more satisfied with the digital meetings than those with a hybrid solution (*M* = 4.23) and those who are at the office (*M* = 3.88). The hybrid group was also more satisfied than the office group. On the downside, 32.8% report more workload during COVID-19. This applies to all groups as there were no gender, age, or worksite differences found; 74% want to work from home more frequently now, as compared to before COVID-19. There are no gender or age differences in this statement, but there is a significant difference between all three worksites [*F*_(2, 558)_ = 64.48, *p* < 0.001, *η*_*p*_^2^ = 0.189] with the highest level among the home-office group (*M* = 4.24), followed by hybrid (*M* = 3.98) and office (*M* = 2.70).

With 36.9% reporting fear of being infected by COVID-19 at work overall, gender [*F*_(1, 555)_ = 6.83, *p* < 0.01, *η*_*p*_^2^ = 0.012] and worksite differences were found [*F*_(2, 558)_ = 9.91, *p* < 0.001, *η*_*p*_^2^ = 0.034]. Women (*M* = 3.06) were more afraid than men (*M* = 2.78), and those with a home office (*M* = 3.11) were more afraid than those working hybrid (*M* = 2.64) and those who were at the office (*M* = 2.59). There was no significant difference in fear between the hybrid and the office group, or between the different age groups. Naturally, 64.3% report that they have less contact than normal with colleagues outside work during COVID-19. This was particularly evident among the young workers (age below 30 years; *M* = 4.04) who agreed significantly more with this statement [*F*_(2, 558)_ = 4.03, *p* < 0.05, *η*_*p*_^2^ = 0.014] than the middle age group (31–50 years; *M* = 3.91) and the oldest age group (51 years or older; *M* = 3.67).

### Evaluation of change due to COVID-19

Seven separate repeated measures ANOVAs were run for all the evaluations of change in the job change variables (Table 5). Box's Tests of Equality of Covariance Matrices were significant for work motivation, work quality, contact with colleagues, and digital competence (*p* < 0.05) indicating a violation of homogeneity of covariance matrices for these variables. Although multivariate test results are robust when you have equal or nearly equal *N*'s in a group (e.g., largest *n*/smallest *n* < 1.5), our ratio with more people having a home office as compared to regular office showed the largest/smallest ratio was 86/17 = 5.06 [which is more than the 1.5 thresholds suggested by Pituch and Stevens (2016)]. It should be noted that when the larger group is associated with smaller variability, the multivariate test becomes too liberal. When the larger group is associated with greater variability, the test becomes too conservative (Pituch and Stevens, 2016). Box's Tests of Equality of Covariance Matrices were non-significant for productivity, concentration, and leader support (*p* > 0.05) indicating no violation of homogeneity of covariance matrices for these variables. With 2 levels of repeated measures, there was no need to conduct Mauchly's test of sphericity as the assumption of Mauchly's sphericity will be met under this situation.

**Table 5:** Means, standard deviations (in italics), and results of repeated measures analyses of variance (MANOVAs) for study variables (evaluation of change due to COVID-19) at time 1 (T1) time 2 (T2) measurement occasion comparing employees having home office, hybrid, or workplace of the employees and who filled in both T1 and T2.

**Job change^a^**	**Home office**		**Hybrid**		**Workplace**		**Within**		**Between**
	**(*****n*** = **86)**		**(*****n*** = **41)**		**(*****n*** = **17)**		**effect**		**effect**
	***T*** **1**	***T*** **2**	***T*** **1**	***T*** **2**	***T*** **1**	***T*** **2**	***η*_*p*_^2^ (time)**	***η*_*p*_^2^(interaction)**	***η*_*p*_^2^(worksite)**
Work motivation	3.15 *(0.79)*	2.94 *(0.86)*	3.12 *(0.56)*	3.00 *(0*.81)	2.94 *(0*.56)	2.76 *(0*.56)	0.05^*^	0.00	0.01
Work quality	3.26 *(0.54)*	3.21 *(0.69)*	3.12 *(0.46)*	3.20 *(0.60)*	3.06 *(0.43)*	3.06*(0.24)*	0.00	0.01	0.01
Work done/productivity	3.35 *(0.73)*	3.48 *(0.81)*	3.27 *(0.59)*	3.17 *(0.59)*	3.18 *(0.64)*	2.82 *(0.81)*	0.00	0.05^*^	0.05^*^
Concentration	3.21 *(0.90)*	3.20 *(0.98)*	3.02 *(0.85)*	3.07 *(0.82)*	2.76 *(0.66)*	2.76 *(0.75)*	0.00	0.00	0.03
Contact with colleagues	2.17 *(0.88)*	2.16 *(0.89)*	2.44 *(0.67)*	2.12 *(0.75)*	2.12 *(0.86*)	1.94 *(0.90)*	0.03	0.03	0.01
Digital competence	3.76 *(0.63)*	3.74 *(0.71)*	3.73 *(0.50)*	3.71 *(0.64)*	3.82 *(0.53*)	3.41 *(0.94)*	0.03^*^	0.03	0.01
Leader support	3.17 *(0.65)*	3.22 *(0.71)*	3.12 *(0.46)*	3.12 *(0.64)*	3.35 *(0.70)*	3.06 *(0.66)*	0.01	0.02	0.01

a The response options ranged from 1 (very reduced) to 5 (significantly increased).

$\eta_{\text{p}}^{2}$ = partial eta squared effects sizes of the interaction of time (i.e., T1, T2) with group (i.e., home office, hybrid, and workplace) on the specific outcome.

* Significant difference.

The multivariate test was significant for the within-person effect of time for work motivation (i.e., Time 1 and Time 2) Wilk's Lambda = 0.953 [*F*_(1, 141)_ = 6.96, *p* < 0.01, *η*_*p*_^2^ = 0.05] yet not for the interaction of time with the worksite. The between-subjects effect was not significant. Thus, motivation significantly decreased between Time 1 and Time 2 for all three worksites. The lack of interaction effect indicates that the variation in the means of motivation over the repeated measurement occasions itself does not vary as a function of group membership (i.e., home office, hybrid, and or office).

The within-person, between-person, and interaction effects of work quality were all non-significant, indicating the work quality did not change across time, or between the worksites.

**Figure 1:**
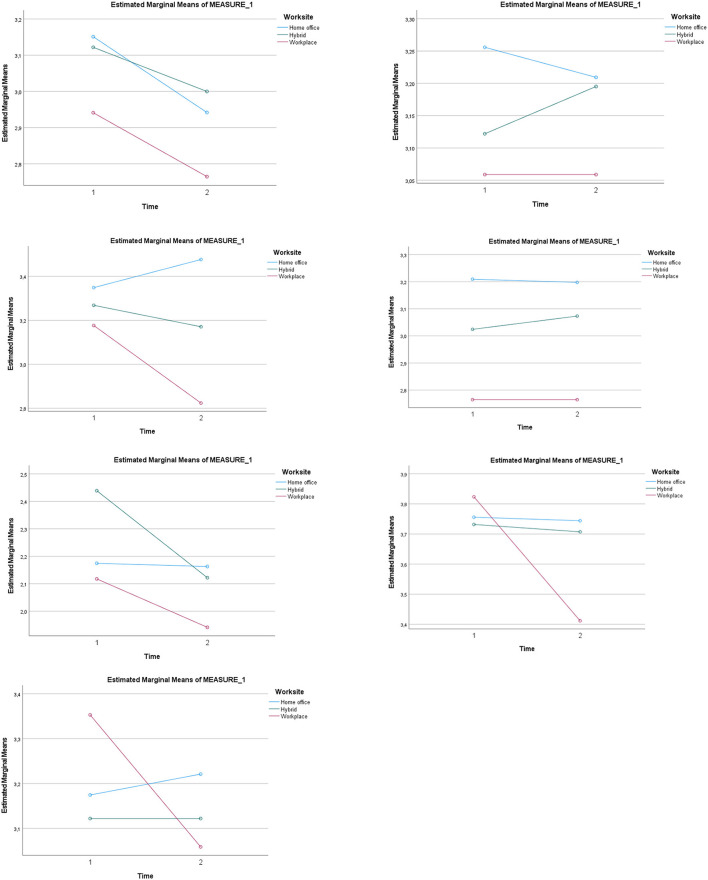
Interaction effects for worksite and time from the seven separate repeated measures analyses of variance for all the evaluation of the job change variables.

Regarding productivity, the multivariate test was not significant for the within-person effect of time (i.e., Time 1 and Time 2) but the interaction of time with worksite was significant with Wilk's Lambda = 0.954 [*F*_(2, 141)_ = 3.40, *p* < 0.05, *η*_*p*_^2^ = 0.05]. Also, the between-subjects effect was significant [*F*_(2, 141)_ = 3.86, *p* < 0.05, *η*_*p*_^2^ = 0.05]. *Post-hoc* analysis with a Bonferroni adjustment revealed significant differences between those having a home office and those who were physically present at the workplace (mean difference = 0.41, *p* < 0.05). Inspection of the profile plots from the interaction effect (see Figure 1) indicates that those having a home office report that they get more work done than the two other groups, but this increases at Time 2 for the home office group and decreases for the group working at the workplace at Time 2.

The within-person, between-person, and interaction effects of concentration were all non-significant, indicating that even though the home-office employees report slightly higher improvements in the levels of concentration at Time 1 and Time 2, they did not significantly differ from the other worksites, and this pattern did not change across time.

Despite a slight decrease in contact with colleagues for all worksites at Time 2, this change was not significant, nor was the difference between the three worksites.

The multivariate test was significant for the within-person effect of time for digital competence (i.e., Time 1 and Time 2) Wilk's Lambda = 0.97 [*F*_(1, 141)_ = 4.38, *p* < 0.05, *η*_*p*_^2^ = 0.05] yet not for the interaction of time with worksite, nor between worksites. While those having a home office or a hybrid solution are quite stable across time, those working at the worksites report that their digital competence decreased at Time 2.

The within, between, and interaction effects of leader support were all non-significant, indicating leader support did not change across time, or between the worksites.

## Discussion

The present study aims to extend our knowledge of telework during a crisis. By using longitudinal data from the COVID-19 pandemic, the aim was 3-fold.

First, we explored reasons for working from home, home-office facilities available, and the desire for working from home now and in the future. There was a considerable change in working situation for the employees as only 3% reported having a homeoffice before COVID-19, whereas 62 and 76.4% reported this to be their main working situation at Time 1 (December 2020) and Time 2 (March 2021) in the study, respectively. This agrees with the situation in Norway at that time. In the autumn of 2020 and the winter/spring of 2021, parts of Norway went into a major social shutdown that hit the big cities particularly hard when infection rates began to rise again. Several companies and service industries had to be closed again, and the widespread use of home offices was reintroduced. In line with this, the quantitative analyses suggested that most agreed that the reason for working at home was to avoid contagion and protect their loved ones from the disease and because it was a requirement from their leader or the government. Few people agreed that this was to get away from the work environment. This indicates that the present findings might be different from previous studies on telework pre-COVID-19, as the motivation to work from home might be more business survival rather than the desire for flexibility and work–life balance (Pirzadeh and Lingard, 2021).

The qualitative comments highlighted some positive aspects of working at home over and above the requirements from the government, with many people reporting it makes everyday life easier. Particularly, less time spent on commuting was mentioned frequently, as well as providing time for family and friends, and restoration. The qualitative comments also suggested that many felt they worked better from home. This relates to efficiency, less disturbance, peace and quiet, better concentration, better technical solutions, more privacy for clients, and a better indoor climate. These findings relate to the quantitative results related to home facilities, suggesting that most agree that they do have access to necessary technical equipment and feel they can work undisturbed at home. Overall, these positive experiences indicate that approximately 8 out of 10 want to be able to work from home more in the future. Future studies should delve deeper into the differences between age, gender, and job position in this preference.

The second aim of the study was to explore participant experience on how COVID-19 has affected their work–life and identify potential group differences in these experiences. Four out of ten agreed that it was hard to distinguish between home life and work during COVID-19. Interestingly, there were no age, gender, or worksite differences in this statement, indicating that this was challenging to the same degree for all participants. This was surprising as previous studies indicate that balancing between work and family life is particularly challenging for women working from home (Eurofound, 2020; Hayes et al., 2021). A gender-by-worksite analysis might have confirmed this statement.

The majority became more positive toward digital solutions (79%), thought that digital meetings functioned well (86.6%), and wanted to use the home office more frequently (74%). This applies especially to those with a home office. There were no gender or age group differences in these statements. The positive attitude regarding digital solutions and home office might relate to positive experiences among those with a home office which has introduced new ways of working. Alternatively, it could be those being the most positive toward digital solutions in the beginning who agreed to work from home during the pandemic.

On the downside, 32.8% reported more workload during COVID-19. This applied to all groups as there were found no gender, age, or worksite differences. This is in line with previous findings suggesting more workload during the pandemic both among frontline workers (Giusino et al., 2021) and teleworkers (Pirzadeh and Lingard, 2021). Yet, this finding departs from another Norwegian study that found significantly more workload among those working from home during the pandemic as compared to the group with no change in their work situation (Da et al., 2022). In the latter study, the workload was measured in general, whereas the present study explored workload related to COVID-19 specifically.

In the present study, women and those with a home office reported significantly more fear of being infected by COVID-19 at the workplace than their counterparts. Overall, almost four out of ten were afraid of COVID-19. The higher prevalence of fear among women aligns with findings suggesting women show greater emotional expressivity in general (Chaplin, 2015) and more specifically with studies showing women to be more distressed and worried than men during the pandemic (Morales-Vives et al., 2020; Al Miskry et al., 2021). However, in contrast to these studies, we did not find any age differences in fear of being infected by COVID-19.

In our study, 64.3% reported that they have less social contact than normal with colleagues outside work during COVID-19. However, there were no gender differences in this statement, nor differences across worksites (home office, hybrid, or workplace). Instead, we found significant differences between the age groups, with the young workers (30 years old or younger) agreeing most with this statement. In a study among Australian construction workers who were required to work from home on alternate weeks, the participants reported that they had been negatively impacted by the reduction in social interaction and connection through work during the COVID-19 pandemic (Pirzadeh and Lingard, 2021). Young workers might be vulnerable to this as they might lack an established network at work and have fewer resources and routines to rely on. This might create a feeling of insecurity and loneliness. Our findings, together with (Microsoft, 2021) predictions that Gen Z is more vulnerable to the impact of isolation due to a higher likelihood of being single, indicate that mental illness among the young should be carefully monitored and prevented.

The third aim of this study was to investigate within- and between-person changes in the evaluation of how COVID-19 has altered different aspects of work across different worksites. In contrast, previous studies suggest that the physical proximity to family members led to increased distractions from work (e.g., interrupting work to address other family members' needs) while working from home (Fukumura et al., 2021). Our study indicates that the home-office group reported the highest change in a positive direction in their level of work motivation, work quality, productivity, and concentration at Time 1. However, these differences across worksites are only significant for changes in self-reported productivity. *Post-hoc* analysis with a Bonferroni adjustment suggested that those with a home office reported significantly more work done (productivity) as compared to those who were physically at the workplace. This aligns with the qualitative findings in our study, suggesting that working from home is more efficient. In addition, the interaction effect of time and group for work amount was significant, indicating that the variation in the means of work amount over the two repeated measurement times itself does vary as a function of group membership (i.e., home office, hybrid, and or office). This is in line with previous findings suggesting that many spent more time on working activities because of the availability of the laptop at home, and having nothing else to do (Pirzadeh and Lingard, 2021). Given that the future of work is predicted to be hybrid, meaning people working from home more frequently, this extended work life might have serious consequences for employees' health and wellbeing. As a response to this, the European Parliament has passed a resolution in favor of the right to disconnect, assuring “worker's right to be able to disengage from work and refrain from engaging in work-related electronic communications, such as emails or other messages, during non-work hours” (European Oberservatory of Working Life (Eurwork), 2021). Nevertheless, it will be interesting to see whether this self-reported productivity continues or diminishes over time. Although getting more work done is highly beneficial for the company, the long-term effect of this extended time working might be harmful. For example, burnout is a condition assumed to develop after a longer period of being exposed to stress at work (Maslach et al., 2001). Moreover, personal proneness to develop an obsessive-compulsive behavior toward work, characterized as workaholism, can eventually lead to an overt expression if employees can work anytime and anywhere (Snir and Harpaz, 2012). Thus, we can only understand the long-term mental consequences of the pandemic as they unfold.

A significant decrease in motivation from Time 1 to Time 2 was also found for all three worksites. This is in line with the Vitamin model (Warr, 2017) suggesting an adaption to the situation. Oksa et al. (2021) also found work engagement to decrease at their last measurement point.

Although the workplace group reported the highest change in digital competence and leader support at Time 1, they report less than the other two groups at Time 2. This decrease was significant for digital competence only. This implies that the change in digital competence remains more stable among the home-office and hybrid groups.

We found no time, interaction, or between-person effects on work quality, concentration, contact with colleagues, or leadership support, meaning these are the same for the different worksites and across time. This suggests it is not necessarily the location of work that reduces contact with colleagues after work, but rather the restrictions provided by the government to socially distance from other people outside your own cohort. It also implies that the quality of work and support from leaders is context-free. Although many reported better conditions for concentration on the job as a reason for working at home in the qualitative and quantitative analyses in this study, the home-office group did not report significantly higher levels of improvement in concentration. Overall, the effects found were small (≤ 0.05), suggesting that COVID-19 has had only minor changes in the work situation for the three worksites over time (Miles and Shevlin, 2001). It should be noted that these findings were done in a Norwegian sample of insurance workers. Although only 3% of this sample had the experience of working from home before the pandemic, indicating a new situation for most of the employees, it has been suggested that the pandemic situation has been less severe in Nordic countries like Norway with less stringent restrictions during the pandemic and a strong welfare model securing the health and wellbeing of the employees (Lilja et al., 2022).

## Limitations and future research

The strength of this study is the longitudinal design and an identification code allowing us to follow the same individuals over time in an extreme and unique situation, allowing us to predict how we will think and act about work and working life in the future (Kniffin et al., 2021). By combining quantitative and qualitative results in this study, it was possible to explore important features of having a home office during the pandemic. Novel situations require new knowledge, and hence other measures to capture the experiences around the situation, in this case, the COVID-19 pandemic's effect on work life. In the present study, we adapted well-known occupational concepts and challenges (i.e., workload, work–home conflict, and leader support) to the experience of COVID-19. In this way, we were able to target the effect of COVID-19 directly and not employee work–home balance in general (i.e., It is harder to balance work and family life during COVID-19). Yet this might preclude some comparisons with other data, but hopefully, it will generate more research and knowledge for future exploration.

By comparing different worksites (home office, hybrid, and workplace) we were able to explore both within- and between-person differences. However, due to the strict regulations of keeping social distance by the government, the majority of participants had a home office (62% at Time 1, and 76.4% at Time 2), making the distribution between the three groups unequal. Thus, the observed effect sizes should be approached with some caution since we may not be able to get a precise estimate of the effects and their effect sizes. With a 3 months interval, some of the non-significant change of time in the present study could be caused by a too short interval between the measurement points. Having more measurement times or different time intervals could have provided a more nuanced picture of how COVID-19 affects the workers' contact with colleagues. Moreover, the self-evaluation of change in the job change variables due to COVID-19 might have precluded some of the effect of time between the two time periods as any evaluation of change reported at T2 might include an evaluation of change felt at T1. Still, the present study found a significant change in motivation and digital competence between T1 and T2.

Working from home was measured by asking employees what best described their working situation, and not by how many days they worked at home. Gajendran's (Gajendran and Harrison, 2007) study suggests number of days working from home matters, with more than 2.5 days a week being beneficial for work–family conflict but harmful for relationships with coworkers. Thus, future studies should explore how many days at home are the most optimal for both employees and employers. Finally, this study was restricted to a specific company in Norway during a challenging time. To test whether certain differences in the constructs are independent of the country of origin of the research, cross-cultural studies are recommended.

## Conclusion

The pandemic has caused a change in work environments, with considerably more people working from home, and with a desire to be able to do so in the future. The flexibility, time saved, and the possibility to spend more time with family and friends are highly appreciated. Our study indicates that those working at home feel they are more productive than before. However, this trend needs to be monitored carefully to avoid work overload and burnout. Although previous studies have indicated more workload and work–family conflict when working from home, our study suggests that this is the same for all gender, age, and work arrangements. Thus, any interventions to ease this burden for the employees would be beneficial for all. As confirmed by the present study, the younger generation seems to be more affected by social restrictions, reporting that they see their colleagues outside work less than normal. As this generation is more likely to be single, making them more vulnerable to the impact of isolation, we urge practitioners, mental health experts, and employers to pay special attention to this age group, especially if they are planning a more frequent use of a home office in the future.

## Data availability statement

The raw data supporting the conclusions of this article will be made available by the authors, without undue reservation.

## Ethics statement

Ethical review and approval was not required for the study on human participants in accordance with the local legislation and institutional requirements. The patients/participants provided their written informed consent to participate in this study.

## Author contributions

SI substantial contributions to the conception or design of the work, or the acquisition, analysis or interpretation of data for the work, drafting the work or revising it critically for important intellectual content, and provide approval for publication of the content. MC contributions to the conception or design of the study, revising the work critically for important intellectual content, provide approval for publication of the content, and provide approval for publication of the content. KG revising the work critically for important intellectual content, contributed to the analyses, and provide approval for publication of the content. CB the development of research questions, revising the work critically for important intellectual content, and provide approval for publication of the content. All authors contributed to the article and approved the submitted version.
